# Distributions of emissions intensity for individual beef cattle reared on pasture-based production systems

**DOI:** 10.1016/j.jclepro.2017.10.113

**Published:** 2018-01-10

**Authors:** G.A. McAuliffe, T. Takahashi, R.J. Orr, P. Harris, M.R.F. Lee

**Affiliations:** a Rothamsted Research, North Wyke, Okehampton, Devon, EX20 2SB, UK; b University of Bristol, Langford House, Langford, Somerset, BS40 5DU, UK

**Keywords:** Beef production, Carbon footprint, Grazing systems, High-resolution data, Life Cycle Assessment, Uncertainty analysis

## Abstract

Life Cycle Assessment (LCA) of livestock production systems is often based on inventory data for farms typical of a study region. As information on individual animals is often unavailable, livestock data may already be aggregated at the time of inventory analysis, both across individual animals and across seasons. Even though various computational tools exist to consider the effect of genetic and seasonal variabilities in livestock-originated emissions intensity, the degree to which these methods can address the bias suffered by representative animal approaches is not well-understood. Using detailed on-farm data collected on the North Wyke Farm Platform (NWFP) in Devon, UK, this paper proposes a novel approach of life cycle impact assessment that complements the existing LCA methodology. Field data, such as forage quality and animal performance, were measured at high spatial and temporal resolutions and directly transferred into LCA processes. This approach has enabled derivation of emissions intensity for each individual animal and, by extension, its intra-farm distribution, providing a step towards reducing uncertainty related to agricultural production inherent in LCA studies for food. Depending on pasture management strategies, the total emissions intensity estimated by the proposed method was higher than the equivalent value recalculated using a representative animal approach by 0.9–1.7 kg CO_2_-eq/kg liveweight gain, or up to 10% of system-wide emissions. This finding suggests that emissions intensity values derived by the latter technique may be underestimated due to insufficient consideration given to poorly performing animals, whose emissions becomes exponentially greater as average daily gain decreases. Strategies to mitigate life-cycle environmental impacts of pasture-based beef productions systems are also discussed.

## Highlights

• Estimates emissions intensity (EI) for each individual cattle on farm.
• Integrates high-resolution data into the life cycle assessment (LCA) framework.
• Proposes an original “uncertainty analysis” based on actual distributions of EI.
• Demonstrates that EI derived by representative animal models may be underestimated.

## 1 Introduction

In order to support the projected global population of 9.15 billion people at mid-century, a 70% increase in total global food production is believed to be required (FAO, 2009) unless drastic measures are taken to improve the global distribution (Ingram, 2011). In the context of livestock production, outputs from meat and dairy enterprises worldwide must be increased by at least 53% and 48%, respectively (Thornton, 2010), and possibly more if the FAO's nutritional recommendations for animal protein are followed to address malnutrition and undernourishment through a balanced diet (FAO, 2014). Worldwide meat production, however, is estimated to generate 7.1 Gt CO_2_-eq of greenhouse gases (GHG) each year, of which cattle contribute 65% (Gerber et al., 2013). Thus, identifying economically and environmentally sustainable methods of beef production is critical to ensure long-term food security (Eisler et al., 2014).

Although feedlot-based beef production systems tend to show a lower level of GHG emissions intensity than pasture-based systems (Pelletier et al., 2010, Peters et al., 2010, Nguyen et al., 2010), they are also known to be the least efficient users of human-edible cereals and legumes in the agri-food industry (Steinfeld, 2006). Pastoral systems for ruminant production, on the other hand, are able to utilise land unsuitable for arable crop production (Eisler et al., 2014, de Vries et al., 2015) by converting forages to valuable sources of protein for humans without driving the food-feed competition for resources (Wilkinson, 2011). Given that the population growth will increase demand for human-edible crops, improving the environmental efficiency of pasture-based beef production systems seems to form, at least for the foreseeable future, part of the solution package for the issue of global food security.

A common method to analyse the trade-offs between economic values of products and environmental damages caused by their production is Life Cycle Assessment (LCA) (Roy et al., 2009). When this method is applied at the farm scale, representative farms are generally constructed from a steady-state herd structure (McAuliffe et al., 2017) or, less frequently, farm surveys (Wiedemann et al., 2015). As information on individual animals is often unavailable, livestock data may already be aggregated at the time of inventory analysis, both across individual animals and across seasons. Although the utilisation of computational tools such as the Monte Carlo method (e.g. Dudley et al., 2014) and the monthly modelling technique (e.g. Brock et al., 2013) allows considerations to genetic and seasonal variabilities in livestock-originated emissions intensity, the degree to which these methods can address the bias suffered by these representative animal approaches is not well-understood.

Using primary data collected on the North Wyke Farm Platform (NWFP) in Devon, UK, this paper proposes a novel approach of life cycle impact assessment that can explicitly account for heterogeneity in animal performance, both individually and seasonally. Field data were measured at high spatial and temporal resolutions, enabling a unique research platform to conduct a detailed analysis of environmental hotspots. The use of individual animal data allowed computation of emissions intensity for each growing calf and, by extension, their intra-farm distributions, offering an alternative method to the Monte Carlo uncertainty analysis whereby livestock performance parameters are assumed to follow distributions pre-identified based on best-available data. The objective of this study, therefore, was to explore the potential benefit of this new approach to consider livestock-originated uncertainty. The research was carried out by means of a case study, in which emissions intensity for pasture-based beef finishing systems at the NWFP was quantified under two methods, namely with and without information on individual animal performance. To the best of our knowledge, this is the first environmental assessment of the English cattle industry based on high-resolution primary data; the industry employs 440,000 people and is estimated to be worth £2.8 billion, with high dependence on grazed systems (Marsh et al., 2012).

## 2 Materials and methods

LCA adhering to the ISO 14040 framework (ISO, 2006), or, in the case of carbon footprint analysis, PAS 2050 guidelines (BSI, 2011), is typically composed of four analytical steps: goal and scope definition, life cycle inventory analysis, life cycle impact assessment and interpretation. This study follows the same protocol to evaluate emissions intensity of pasture-based cattle production systems. However, as the study only entails the post-weaning (finishing) stage of the cattle lifecycle (for reasons discussed below), it is not a full carbon footprint analysis in the strictest sense as defined by PAS 2050.

### 2.1 Study site

The NWFP is located in Devon, a southwest county of England, UK (50°46′10″N, 3°54′05″W) and consists of three hydrologically isolated small-scale (21 ha) livestock farms known as “farmlets” (Fig. S1). Each of the three farmlets operates under a different pasture management system, with swards of: (1) permanent pasture (PP), of which no field has been reseeded for at least 20 years; (2) white clover (*Trifolium repens*)/high sugar perennial ryegrass (*Lolium perenne*) mix (WC), which aims to maintain 30% ground cover by white clover; and (3) high sugar perennial ryegrass monoculture (HS), which utilises the latest improved grass varieties.

Every autumn, 30 Charolais x Hereford-Friesian calves enter each farmlet at the point of weaning. At this time, animals are blocked between sexes and then randomly allocated to the farmlets from an adjacent but separate cow-calf operation, of which grasslands are permanent pasture similar to the PP system. After entering the NWFP, animals are typically housed from October to April to avoid destruction of soil structure during the wet season, then moved and kept outdoors on their respective pastures until they reach target weights of ca. 555 kg for heifers and 620 kg for steers and estimated meat quality scores (RPA, 2011) of “R” (conformation) and “4L” (fat). If animals do not meet these finishing criteria at pasture, a second housing period may be required. Throughout housing periods, animals are fed silage comprising grasses and legumes harvested from their own allocated systems (PP, WC or HS). While the NWFP's general principle is to finish cattle solely off pasture and silage, depending on the quantity and quality of silage produced in any particular year, strategic supplementary feed to balance energy and protein demands may be used and recorded. When strategic feeding occurs, its quantity is set at a uniform rate across animals to minimise confounding effects. Cattle are housed in barns deep-bedded with barley (*Hordeum vulgare*) straw, and farmyard manure (FYM) produced is stored temporarily in middens until spreading in the next spring following first silage cut. The duration of this storage was assumed to be six months in the model below.

Data collection on the NWFP began in 2011, when it was established as a UK national capability programme supported by Biotechnology and Biological Sciences Research Council (BBSRC). Prior to 2013, all three farmlets were composed of permanent pastures largely (>60%) dominated by perennial ryegrass. Between 2013 and 2015, the WC and HS farmlets were reseeded with white clover and high sugar perennial ryegrass, with the choice of cultivars based on the national recommendation list of latest germplasm (BGS, 2013). Throughout this transition period, the WC and HS fields underwent ploughing, ring rolling, harrowing, herbicide spraying, drill seeding and flat rolling; the PP system remained unaltered (Table S1). On crop establishment, the HS (and PP) pastures received standard N, P and K fertilisation, whereas the WC fields received a lower amount of N, predominately in the form of FYM. Soil tests were conducted to assess the quality of the land post-ploughing. The WC and HS soils were found to be generally acidic, resulting in the application of lime to neutralise the acidity at variable rates between 150 kg/ha and 725 kg/ha. In addition, the WC system was found to be low in P levels, and consequently required higher levels of P_2_O_5_ application than the other two systems. Further information on the NWFP's design concept and operation, including details of the transition process, is provided elsewhere (Orr et al., 2016).

### 2.2 System boundary and functional unit

**Fig. 1:**
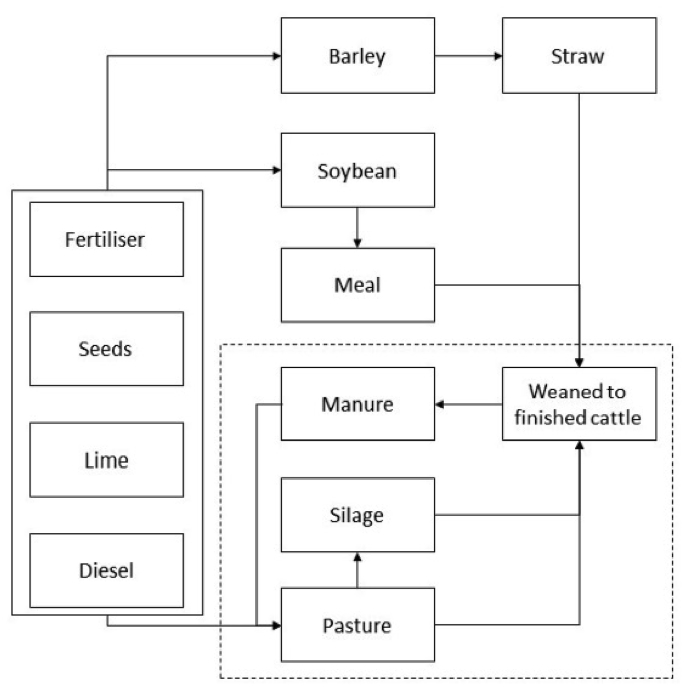
System boundary of the present study. The dashed line represents the North Wyke Farm Platform.

A schematic diagram of the system boundary is provided in Fig. 1. The present study adopts a “gate to gate” approach (Berton et al., 2016, Ogino et al., 2004), whereby all non-capital inputs and outputs related to the post-weaning phase of cattle production are included in the model. Production processes for farm building infrastructure were excluded from the model following the approach adopted by recent studies (Buratti et al., 2016, Mogensen et al., 2015). Temporally, the present study follows emissions intensity of 90 cattle (30 per farmlet) that were born in the spring of 2014. Thus, the on-farm component of Fig. 1 corresponds to the period from October 2014, when they were weaned from their mothers, to their departure to the slaughterhouse, around December 2015 for the majority of the animals, on meeting weight and carcass specification targets.

While it is acknowledged that cow-calf operations may generate as much as two-thirds of the total carbon footprint associated with beef production, particularly on pasture-based systems (Pelletier et al., 2010), the suckler system was excluded from the present analysis for three reasons. First, by randomising the allocation of calves to each farmlet at weaning, factors related to *mothers'* body conditions that affect *calves’* performance early in life (e.g. quantity and quality of milk provided) are also randomised, and thus the difference in system-wide economic and environmental performance amongst the three finishing systems becomes fully attributable to their pasture management strategies. Second, as all animals are maintained on the same (external) permanent pasture system prior to their entrance to the NWFP, excluding this stage from the computation of lifecycle emissions intensity does not affect the relative ranking of the three systems. Third, as the cow-calf operation in North Wyke is not part of the NWFP, it does not record field data at a resolution comparable to what is collected on the NWFP. While it is possible to estimate emissions intensity of the suckler operation based on a combination of low-resolution data and published equations, doing so would likely compromise the accuracy of the methodological comparison between the existing approach based on a representative animal and the individual animal approach proposed by the present study (to be outlined below), and therefore was judged to be undesirable.

Given that the entire lifecycle of cattle was not examined, the more common functional units for beef LCA studies, such as liveweight (Ridoutt et al., 2011) or carcass weight (Mogensen et al., 2015, Peters et al., 2010) were inappropriate. Instead, the functional unit was set as “1 kg of liveweight gain (LWG)”, a key indicator of the animal performance post-weaning that has previously been adopted by Casey and Holden, 2006, Dick et al., 2015 and Ruviaro et al. (2015), amongst others. The use of this functional unit implies that the partitioning of an additional mass acquired by livestock (between muscles, fats and other parts of the body) is assumed not to differ considerably amongst individual animals.

### 2.3 Inventory analysis and impact assessment

As discussed, the majority of on-farm information utilised in the present study was collected in the form of primary data. Individual animals were weighed every two to four weeks using a cattle crush and weigh head, providing a high temporal resolution for average daily gains (ADG). During the grazing season, sward snip samples were collected in the same weeks when animals were weighed from all fields occupied by cattle at that time. These samples were cut at grazing height (5 cm above ground level) along a W-transect, ignoring dead material, seed heads and weeds animals tend to avoid. During winter, grab samples of silage were collected at a similar frequency, from five points along the width of each barn during feeding time, so that they represented roughage consumed by livestock at that time. Samples were stored at −20 °C until chemical analysis was carried out.

Modified Acid Detergent Fibre (MADF) composition for both pasture and silage samples was quantified using a FOSS Fibertec 8000 Auto Fiber Analysis System following the method of Clancy and Wilson (1966). Samples were freeze dried and then ground using a Cyclone Sample Mill so that material could pass through a 1 mm sieve. Following this preparation, 1000 ± 2 mg of sample was added to oven dried crucibles. Crucibles were first inserted to the Fibertec cold extraction unit to remove excessive fat content using 25 ml of acetone and then placed into the Fibertec hot extraction unit. Acid detergent solution (ADS) was made by mixing 0.5 M (1N) of H_2_SO_4_ with 20 g/l of CTAB (HexadecylTrimethylAmmonium Bromide 98%). Using this solution, modified acid detergent solution (MADS) was subsequently produced by mixing equal volumes of ADS and H_2_SO_4_. The hot extraction unit automatically distributed MADS and antifoaming agent (n-Octanol) to the samples. Following hot extraction, 25 ml of acetone was added to samples and drained. Once analysis was complete, the derived MADF fractions were converted to corresponding metabolisable energy (ME) values using equations independently calibrated for UK pastures and silages (Alderman and Cottrill, 1993). These values were further converted to digestible organic matter content (DOMD; reported as digestible energy) using a separate equation (Alderman and Cottrill, 1993).

Total N contents of feed were measured using an elemental analyser and isotope ratio mass spectrometer. Samples were weighed to 2 ± 0.1 mg using a Mettler Toledo MX5 electronic microbalance and inserted to 5 × 3.5 mm tin capsules. They were then analysed in a Carlo Erba NA2000 elemental analyser connected to a Sercon 20–22 isotope ratio mass spectrometer. The derived total N values were converted to crude protein content by multiplying the standard coefficient of 6.25 (FAO, 2003).

Detailed records of all farm inputs were maintained throughout the season. These include, for example, the type and amount of fertilisers and pesticides used, the areas these products were applied to, and supplementary feeds used during housing. Table 1 provides a detailed breakdown of inputs applied to the NWFP during the temporal boundary of the study. Background processes such as production of fertiliser, supplementary feeds, bedding and seeds, were sourced from the *ecoinvent* database V3 (Wernet et al., 2016). Sea-based transportation distances were calculated using data from Portworld (2016), while road distances were calculated using a geographical information system (GIS) platform.

**Table 1:** Inventory of material inputs for each system.

Variable	Unit	PP^d^	WC^e^	HS^f^
Area	ha	21.61	20.85	21.45
Fertiliser area	ha	21.24	20.52	21.03
FYM^a^ area	ha	18.90	17.97	18.34
Yield	kg DM/ha	11474	10780	10417
Fertiliser applied				
N	kg	4951	681	4346
P	kg	206	1125	208
K	kg	554	454	312
Lime	kg	0	3002	4361
FYM^a^	t	118	118	98
Pesticides				
Glyphosate	kg	0	7.51	15.25
Fluroxypyr	kg	0	0	0.98
Seeds				
Grass	kg	0	734	650
Clover	kg	0	42	0
Diesel for machinery	l	342	1181	1295
Soybean	kg	651	651	672
Straw	kg	38920	39894	39685
Transport				
Soybean (sea)	tkm	6267	6267	6469
Soybean (road)	tkm	155	155	160
Straw (road)	tkm	2436	2497	2484
Fertiliser (road)	tkm	2444	2252	3949
Pasture quality				
DE^b^	%	77.55	77.7	76.78
CP^c^	%	20.72	20.12	17.41
Silage quality				
DE^b^	%	65.76	64.05	64.66
CP^c^	%	11.44	9.24	11.92

a FYM: farmyard manure.

b DE: digestible energy.

c CP: crude protein.

d PP: permanent pasture.

e WC: white clover/high sugar grass mix.

f HS: high sugar grass monoculture.

**Fig. 2:**
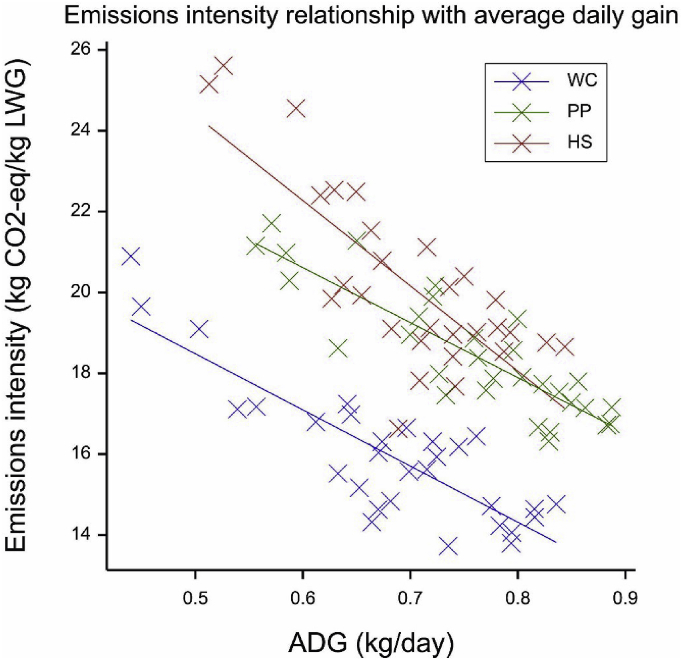
Relationship between global warming potential (GWP) and average daily gains (ADG) under each system. PP: permanent pasture; WC: white clover/high sugar grass mix; HS: high sugar grass monoculture.

**Fig. 3:**
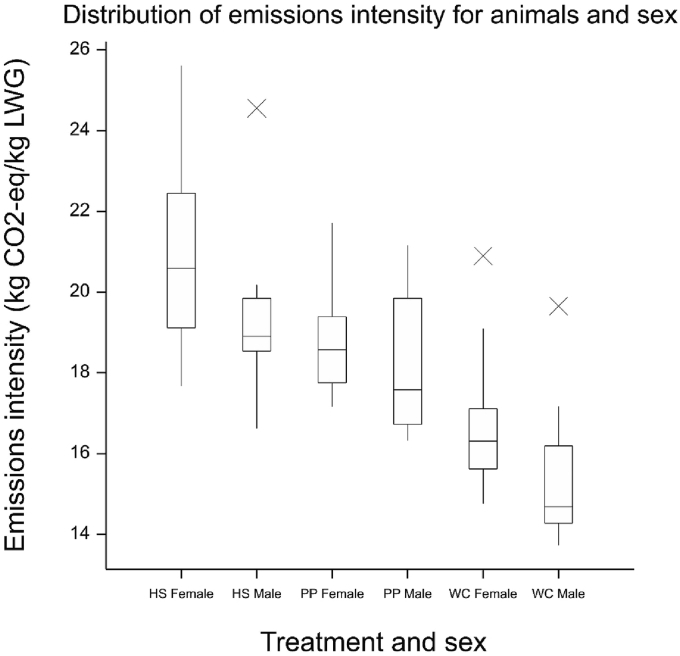
Distribution of global warming potential (GWP) per animal by sex. Outliers located further than 1.5 times the interquartile range beyond the quartiles are each denoted with a cross (×).

Emissions arising from livestock and pastures were calculated using a modified IPCC Tier 2 approach (IPCC, 2006). In order to examine both temporal differences of emissions and the effects of animal heterogeneity, livestock emissions were calculated for each animal for each time period (i.e. between two weighing events) using the weighing records and digestible energy and crude protein values obtained in the methods described above. Calculations were programmatically automated and linked to the NWFP database so as to apply different parameters depending on the animal's age, location and feed being consumed (***Data in Brief* article**: Fig. 2, Fig. 3). This model design was motivated by an earlier finding that the difference in direct emissions between times when animals are on pasture and in housing is primarily driven by digestibility (affecting rumen methane production) and, to a lesser degree, crude protein content (affecting nitrogen-based emissions) of feed (Boadi and Wittenberg, 2002).

On the NWFP, sheep also occupy grassland as part of rotational grazing systems, although they do not share the same pasture with cattle at any given time (Orr et al., 2016). Considering that their manure also contributes to pasture growth (and thus indirectly facilitates cattle LWG) and *vice versa* (cattle manure facilitates sheep LWG), the entire environmental burdens originating from pastures were first split between the two enterprises based on economic values of products leaving the system boundary (i.e. economic allocation). The emissions allocated to the cattle operation were further split among individual animals under the rules that: (a) emissions originating from material inputs to pastures (e.g. inorganic fertilisers and use of machinery) and sheep manure were evenly distributed across 30 cattle on each system; and (b) emissions arising from cattle manure (minus those allocated to the sheep enterprise) were calculated individually for each cattle, taking animals’ growth performance into consideration. All results reported below are net of GHG emissions attributable to sheep production.

Grasslands in the southwest of England are typically located on hilly land with soils that become supersaturated. As these lands are unsuitable for arable crop production, emissions owing to land use and land use change were not included in the present model. Similarly, given the small quantities of soybean (*Glycine max*) supplemented to the animals during the final weeks in housing (Table 1), land use change (LUC) associated with the production of soybean was not considered. Finally, grasslands are sometimes credited as being net sinks of CO_2_, although a large degree of uncertainties exists for these estimates (Beauchemin et al., 2011). Following PAS 2050 guidelines (BSI, 2011), the potential effect of changes in soil carbon stock on emissions intensity was not considered in this study, as reseeding of two treatments (WC and HS) did not involve LUC. For the purpose of calculating environmental burdens associated with on-farm activities for reseeding (Table S1), WC and HS systems were assumed to be renewed every five years. As will become clear, the results were not sensitive to this sowing interval.

On completion of the life cycle inventory, emissions intensity for each individual animal was estimated according to the IPCC (2013) 100-year average method using SimaPro V8.2.3 (PRé Consultants, 2016). CH_4_ and N_2_O were respectively assumed to have 28 and 265 times greater impacts than CO_2_ on climate change. Processes were designed so the sum of emissions from all individual animals theoretically equates to the total emissions from the cattle-finishing enterprise of each farmlet.

### 2.4 Interpretation

Statistical interpretation was carried out using GenStat V17.1 (VSN International, 2014). Based on performance data and emissions intensity estimates for individual animals, multi-sample F-tests (one-way analysis of variance) and two-sample t-tests were conducted to examine differences in livestock performance and emissions intensity between the farmlets. Correlations between emissions intensity and its potential determinants were assessed using Pearson's correlation coefficient.

As discussed, estimation of emissions intensity for individual animals in this study was motivated by uncertainty inherent within life cycle data, which is regarded as one of the most limiting factors of the LCA framework (Groen and Heijungs, 2017). However, information on the performance of individual animals is not always available to LCA practitioners, especially outside the research farm environment. In order to examine the potential discrepancy in model outputs between these two situations, an alternative method of estimation was also set up as part of the analysis. Here, variables related to animal performance (e.g. ADG and days on farm) were averaged across the entire herd based on low temporal resolution (yearly) data, generating a single value of emissions intensity for a representative animal reared on each farmlet. Following this procedure, the effect of uncertainty was evaluated by means of a Monte Carlo analysis, and the resultant outputs were compared pairwise between the three farmlets. Furthermore, to evaluate the degree of interactions between the two methods of uncertainty analysis, a similar assessment was also carried out for the best and worst performing animals on each farmlet (as judged by emissions intensity) that were identified under the original approach. All Monte Carlo simulations were conducted using SimaPro V8.2.3 (PRé Consultants, 2016), where parameters were randomly drawn over 1000 iterations from the distributions summarised in Table S2.

Finally, in line with ISO 14040 (ISO, 2006), a sensitivity analysis was conducted to test the effect of choosing the economic allocation method (for emissions from pastures) on the model outputs. The mass allocation approach was selected as an alternative, whereby the allocation ratio was determined by estimated dry matter intake (DMI) of cattle and sheep. In addition, a sensitivity test was also carried out to test the impact of having applied the IPCC 2013 conversion factors to derive CO_2_-equivalent values for other GHG (28 for CH_4_ and 265 for N_2_O) *vis-à-vis* the superseded IPCC 2007 factors, which had considerably different specifications (25 for CH_4_ and 298 for N_2_O).

## 3 Results and discussion

### 3.1 Inter-system differences

Across the three systems, a short-term decrease in ADG was observed immediately post-weaning. As animals grew larger, their ADG increased to around 1.4–1.6 kg/d, until they reached a mature age and then slowed down to “finish” or meet conformation and fat scores (***Data in Brief* article**: Fig. 1). The relatively low overall ADG compared to the common target rate in the study region (0.8–1.0 kg/d) was due to extended housing and difficulty in satisfying carcass specification criteria. A statistically significant difference in ADG was observed amongst the three systems, with the animals on PP growing faster than WC and HS (Table 2). This result is largely attributable to their relative performance during the aforementioned conditioning period, while the degree of inter-system difference was considerably lower earlier in the season. As a result of randomised allocation and weight targeting, there were no significant inter-group differences for entry weight or finishing weight.

**Table 2:** Livestock performance under each system.

Parameter	Unit	PP^a^	(SD^b^)	WC^c^	(SD)	HS^d^	(SD)	*p*-value^e^
Entry weight	kg	279	(32.08)	279	(28.76)	284	(35.76)	0.80
Finishing weight	kg	607	(50.75)	582	(47.15)	590	(39.23)	0.12
Total growth	kg	328	(41.68)	304	(45.73)	307	(38.67)	0.05
Time on Farm Platform	d	448	(40.33)	461	(43.68)	453	(31.97)	0.46
Average daily weight gain	kg/d	0.76	(0.10)	0.68	(0.10)	0.70	(0.08)	<0.01

a PP: permanent pasture.

b SD: standard deviation.

c WC: white clover/high sugar grass mix.

d HS: high sugar grass monoculture.

e Based on multi-sample F-tests.

Table 3 displays the major contributors to total emissions intensity in each farmlet. In consonance with other LCA studies on beef production systems (de Vries et al., 2015), methanogenic emissions from the rumen were the single greatest source of GHG emissions irrespective of pasture management strategies. The WC system had the lowest average emission intensity across all animals (16.0 kg CO_2_-eq/kg LWG), a result primarily driven by lower requirements of inorganic N fertiliser, followed by PP (18.5 kg CO_2_-eq/kg LWG) and HS (20.2 kg CO_2_-eq/kg LWG). Multi-sample F-tests based on emissions intensity of individual animals showed there were significant differences across the three treatments (*p* < 0.001). Pairwise (via t-tests), emissions intensity for WC was significantly lower than PP (*p* < 0.001) and HS (*p* < 0.001), while PP was significantly lower than HS (*p* < 0.001). With regard to direct livestock emissions, the PP farmlet performed most favourably due to higher ADG (Table 2). However, care should be taken at the interpretation of ADG; relatively low nutrient values for WC (crude protein) and HS (digestible energy) could be a reflection of the fact that WC and HS swards were close to establishment (Table 1). Higher animal performance on PP notwithstanding, reduced N fertiliser usage on WC was by far the greatest saving to total GHG emissions across all systems although, over the long term, the negative impact of legumes on the soil carbon stock (Herridge and Brock, 2016) may also need to be considered.

**Table 3:** Factors contributing to emissions intensity of individual cattle. Results are presented as the average value across 30 cattle assigned to each system in the unit of kg CO_2_-eq/kg LWG.

Source	PP^c^	(Range)	WC^d^	(Range)	HS^e^	(Range)
Enteric fermentation (CH_4_)	7.09	(6.16–8.02)	7.7	(6.43–9.70)	7.52	(5.24–9.61)
Manure management (CH_4_)^a^	1.36	(0.73–1.78)	1.83	(1.11–2.56)	1.68	(1.27–2.59)
Manure management (direct N_2_O)	1.15	(0.99–1.34)	1.06	(0.66–1.38)	1.06	(0.70–1.32)
Manure management (indirect volatilisation N_2_O)	0.2	(0.17–0.22)	0.18	(0.11–0.23)	0.18	(0.12–0.23)
Barley production	0.56	(0.44–0.69)	0.62	(0.46–0.90)	0.61	(0.50–0.87)
Ammonium nitrate production	3.56	(2.78–4.39)	0.53	(0.39–0.76)	3.32	(2.73–4.72)
Fertililser application (N_2_O)	2.03	(1.59–2.50)	0.3	(0.23–0.44)	1.89	(1.56–2.69)
Urine and dung from ewes on pasture (N_2_O)	0.6	(0.47–0.74)	0.67	(0.50–0.97)	0.66	(0.54–0.94)
Farmyard manure application (N_2_O)	0.43	(0.25–0.55)	0.45	(0.30–0.62)	0.52	(0.40–0.78)
Crop residues (N_2_O)	–	–	0.33	(0.25–0.48)	0.33	(0.27–0.46)
Indirect emissions from leaching (N_2_O)	0.2	(0.17–0.24)	0.11	(0.08–0.14)	0.2	(0.15–0.29)
Urine and dung from cattle on pasture (N_2_O)	0.25	(0.19–0.31)	0.21	(0.13–0.29)	0.19	(0.11–0.27)
Single superphosphate production	0.03	(0.02–0.04)	0.18	(0.14–0.27)	0.03	(0.03–0.05)
Others^b^	1.03	(0.80–1.26)	1.8	(1.34–2.58)	1.97	(1.61–2.80)
Total	18.47	(16.32–21.71)	15.96	(13.73–20.90)	20.17	(16.63–25.61)

a Methane arising from manure management was calculated under a deep bedding system assuming a methane conversion factor of 20% and an average annual temperature of 12 °C.

b Includes processes which account for <1% of the total emissions intensity: lime production and decomposition, soybean production, pesticide production, transportation and diesel combustion for machinery.

c PP: permanent pasture.

d WC: white clover/high sugar grass mix.

e HS: high sugar grass monoculture.

Fig. 2 displays the relationship between ADG and emissions intensity under each treatment. Strong and statistically significant negative correlations were found between the two variables for all three systems (*r* = −0.86, −0.84 and −0.77 respectively for PP, WC and HS; all *p* < 0.001), suggesting that the inter-system differences in mean emissions intensity values are, to a large degree, explained by differences in ADG. As for the reasons for differences in ADG, the slower growth rates by WC cattle can largely be attributed to the lower crude protein content in silage (Table 1). For HS animals, the greater heterogeneity of the monoculture swards at the height of the pasture growing season, which was consistently observed and in this instance resulted in a lower yield recorded from summer silage cuts (Table 1), would likely have been a leading contributing factor, although the digestible energy of HS grass was also lower than expected (but statistically not different to PP). This hypothesis, in turn, seems consistent with smaller variances for both ADG and emissions intensity amongst PP animals, as seen in both Table 3 and Fig. 2. The above evidence indicates that, while the PP system has a higher emissions intensity than the WC system on average, it may possess a comparative advantage from the viewpoint of system stability and thus a less stringent requirement for animal selection, at least during early years of sward establishment following pasture renewals. To maximise the genetic potential of latest germplasm used by the WC and HS farmlets, strategies to reduce spatial variability of swards, such as spatial separation (Sharp et al., 2014), overseeding (Rouquette, 2016) and precision agriculture (Hedley, 2015), may need to be explored. This is so because pasture designs that optimise the balance of nitrogen and energy release in the rumen, both at grazing and from silage, will increase ruminal microbial protein synthesis and subsequently animal performance (Lee et al., 2001, Merry et al., 2006).

### 3.2 Intra-system distributions

As the emissions associated with pasture were evenly distributed across 30 cattle on each farmlet, the observed intra-system variation in emissions intensity is solely attributable to individual livestock performance. A closer investigation of these distributions suggests that, although emissions from livestock were not the primary drivers of relative emissions intensity amongst different farmlets, individual animal heterogeneity played a key role in distributions of emissions intensity within each particular farming system (Fig. S2). In addition to 33% (PP), 52% (WC) and 54% (HS) differences in emissions intensity between the best and worst performing animals on the farms (Table 3), there were notable differences in animal performance between sexes under two of the three treatments (Fig. 3). Steers from the WC farmlet were found to have a significantly lower emissions intensity than WC heifers (difference in means = 1.4 kg CO_2_-eq/kg LWG; *p* = 0.020 based on the paired *t*-test). Similarly, HS males had a significantly lower emissions intensity than HS females (1.7 kg CO_2_-eq/kg LWG; *p* = 0.027). While there were no significant differences in ADG between the sexes within either of these treatments, male cattle had higher total LWG than females for both WC and HS systems (*p* = 0.033 and *p* = 0.037, respectively). Interestingly, HS heifers spent significantly less time on the NWFP than steers (difference in means = 34 days; *p* = 0.010) because of their lower target weight and propensity for females to meet carcass specification requirements more easily. However, the associated savings in livestock-based emissions were not large enough to offset the benefits of larger total growth by steers. This finding reiterates the importance of considering interlinkages with external supply chains (Brock et al., 2013) and may support an argument for dairy beef production (in which more males are reared for meat than females) to create more sustainable livestock systems (de Vries et al., 2015), although the slower growth rate by dairy breeds, as well as the greater finishing potential of bulls, must also be taken into consideration in this debate. Further research is required before drawing any conclusion regarding the optimal interlinkages between beef systems and dairy systems, which is beyond the remit of the present study.

### 3.3 Methodological comparisons

As described earlier, emissions intensity was also computed for a pre-averaged representative animal on each farmlet. The resultant point estimates for emissions intensity under PP, WC and HS systems were 17.6, 14.3 and 18.8 kg CO_2_-eq/kg LWG, respectively. Compared to the arithmetic means of emissions intensity values across individual animals (Table 3), the alternative approach was found to underestimate the emissions intensity by 0.9–1.7 kg CO_2_-eq/kg LWG, or up to 10% of system-wide emissions.

According to Monte Carlo pairwise comparisons carried out for these pre-averaged animals, the PP and HS systems both had significantly higher emissions intensity than the WC system (*p* = 0.017 and *p* = 0.001, respectively); however, there were no significant differences between the PP and HS systems (*p* = 0.293). This finding contrasts with the aforementioned *t*-test results based on emissions intensity derived for individual animals, whereby the mean values from all three systems were found to be significantly different. The reason behind this discrepancy is thought to be the muting effect held by averaging herd statistics on the extreme animals. In other words, representative animal approaches fail to sufficiently consider burdens arising from poorly performing animals, whose emissions intensity becomes exponentially (as opposed to linearly) higher as their ADG nears zero; this results in “empty” methanogenic emissions to merely sustain, rather than increase, their bodyweights. Indeed, the upper limit values of the 95% confidence intervals estimated by the Monte Carlo method were found to be universally smaller than the emission intensities derived for the worst-performing “real” animals, and considerably so for the WC and HS systems under which ADG tended to be more variable (Table S3). This result is rather surprising given that the parameter distributions adopted for the present Monte Carlo simulations were designed to represent multiple sources of uncertainties including, *but not limited to*, animal heterogeneity.

**Fig. 4:**
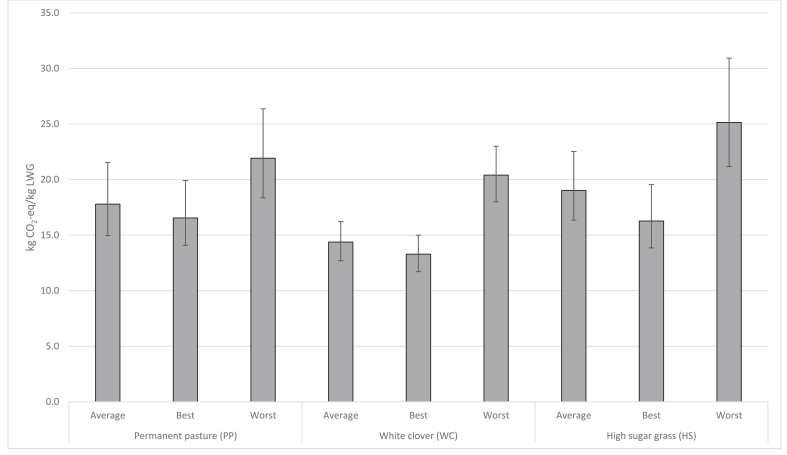
Results of Monte Carlo simulations applied to pre-averaged representative animals and the best and worst performing animals. Error bars represent 95% confidence intervals.

Fig. 4 depicts the above Monte Carlo results diagrammatically, alongside the uncertain ranges derived for the best and worst performing animals on each farmlet. Here, the hypothesis about the importance of considering the “weakest” animals seems to gain further credibility, as the worst performing animals demonstrate far wider 95% ranges than the average (and best performing) animals—especially for the WC system, whose overall burdens are more strongly affected by livestock emissions due to its lower ammonium nitrate application rates. While the ranges given for the best and worst performing animals may be slightly overestimated compared to those given for the average animals (as the animal heterogeneity has been considered twice, first in the form of animal performance and then through parameter distributions), this condition applies equally to both the best and worst animals; it would be reasonable to conclude, therefore, that the use of pre-averaged data *may* result in underestimation of emission intensities, both in terms of point estimates as well as the width of confidential intervals. It should be noted, however, that this finding has only been drawn in the context of farm-scale LCA, and its relevance at the regional and national scales is not necessarily straightforward.

### 3.4 Sensitivity analysis

The results of the sensitivity analysis for allocation methods showed that the ratios derived for mass allocation of pasture-originating burdens did not deviate from the values originally prepared for economic allocation in any considerable manner. On average across the three systems, emissions assigned to cattle were 78% under economic allocation (i.e. 22% assigned to sheep) and 72% under mass allocation. This finding offers an interesting insight about the livestock market in the UK that the economic values of livestock products are strongly correlated with the amount of feed required to produce them. As a result, emissions intensity for WC, PP and HS, respectively, decreased by 2%, 3% and 4% under mass allocation of pasture, suggesting that modelling results were robust to the allocation method adopted.

Using the old IPCC 2007 conversion factors was found to affect model outputs, with both the mean emissions intensity for WC (14.3 kg CO_2_-eq/kg LWG) and HS (18.9 kg CO_2_-eq/kg LWG) resulting in significantly lower emissions intensity (*p* < 0.001 and *p* = 0.027). There was, however, no significant difference for the PP system, a result caused by lower CH_4_ emissions from the system as a consequence of higher ADG. Since applying different conversion factors can significantly alter the results of a study, this adds another dimension to the already challenging cross-comparability issue in LCA research (McAuliffe et al., 2016, McAuliffe et al., 2017). Future studies should be mindful that their estimate of emissions intensity may be higher than older work that employs the 2007 coefficients not because of the inefficiencies of farming strategies but because of the different assumptions adopted, particularly if the systems in question operate under low animal productivity.

### 3.5 Wider implications for pasture-based beef production systems

The findings from the present study have highlighted that considerable opportunities still exist to improve the environmental performance of pasture-based beef production systems without compromising the economic sustainability of commercial farms. These channels include innovative use of legumes, adoption of “low carbon” inputs, and careful selection of both pasture and animals based on genetics and performance. While emissions intensity of efficiently produced grass-fed beef would remain higher than the majority of grain-fed beef, pork and chicken (de Vries and de Boer, 2010), other facets also need to be considered in the holistic debate on global food security. First, as already discussed, grazing animals compete less with humans for land resources that are suitable for arable production and thus economically more valuable (van Zanten et al., 2016, Van Kernebeek et al., 2016), resulting in more favourable land value-to-protein conversion efficiencies (de Vries et al., 2015, O'Mara, 2012). Second, when maintained at an appropriate stocking density, livestock often contribute positively to long-term soil fertility (Haynes and Williams, 1993), provision of ecosystem services (Dick et al., 2016, Horrocks et al., 2014), and more generally the sustainability of grasslands and rangelands. Third, contrary to common belief, beef finished on pasture when consumed in moderation does not pose health risks caused by high saturated fat content and high omega-6 to omega-3 ratios (n-6:n-3); when adipose tissues are removed from steak produced from pasture-raised cattle, the resultant meat often carries n-6:n-3 of less than 3 (Warren et al., 2008), a value within the recommended range for human nutrition (Simopoulos, 2006). In contrast, beef finished off concentrates typically records n-6:n-3 greater than 12 (Warren et al., 2008).

As a recent study points out (Coelho et al., 2016), these aspects, and in particular the final point regarding human nutrition, have largely been overlooked by the existing literature discussing the “shadow” of livestock production (Sabaté et al., 2015, Steinfeld, 2006). Our future work will build upon the present study by examining the nutritional value of meat produced from each individual animal and incorporate this information into an environmental assessment framework, with the view to define the role of livestock production systems in global food security based on high-resolution data and a clear and unbiased methodology.

## 4 Conclusion

This study used two approaches to calculate the partial carbon footprint of three pasture-based beef-cattle finishing systems trialled on the NWFP. In the first approach, emission intensities were calculated for individual animals, whereas pre-averaged livestock data were utilised in the second approach. The results suggested that the outputs derived from pre-averaged data may be underestimated due to insufficient consideration given to poorly performing animals. The systematic bias identified by this study calls for careful interpretation of potentially optimistic LCA results based on pre-averaged herd data. At the same time, it opens up a large opportunity to reduce carbon footprints associated with livestock production systems, as the environmental benefit of evidence-based animal selection is likely to be considerably larger than currently thought. Future studies should further investigate policy implications of the present finding, including its applicability to regional and national-scale analyses.

## Appendix A Supplementary data

mmc1 mmc1
