# Supplementary material for: Distributions of emissions intensity for individual beef cattle reared on pasture-based production systems
Source: J Clean Prod. 2018 Jan 10;171:1672–80. doi: 10.1016/j.jclepro.2017.10.113 (PMC5738973; doi:10.1016/j.jclepro.2017.10.113)
Supplement: mmc1 [file mmc1.pdf]

Distributions of emissions intensity for individual beef cattle reared on pasture-based production systems

G.A. McAuliffe[1,2], T. Takahashi[1,2]*, R.J. Orr[1], P. Harris[1] and M.R.F. Lee[1,2]

[1] Rothamsted Research, North Wyke, Okehampton, Devon, EX20 2SB, UK,

[2] University of Bristol, Langford House, Langford, Somerset, BS40 5DU, UK

* Corresponding author. Tel: +44 1837 883558. Email: taro.takahashi@rothamsted.ac.uk

Supplementary material (6 pages)

Figure S1 Map of the North Wyke Farm Platform in Devon, UK

Figure S2 Distribution of emissions intensity per animal under each system

Table S1 Farm activities carried out under each system

Table S2 Distributions of uncertainty parameters assumed in Monte Carlo simulations

Table S3 Comparison of emissions intensity (kg CO2-eq/kg LWG) derived under two methods

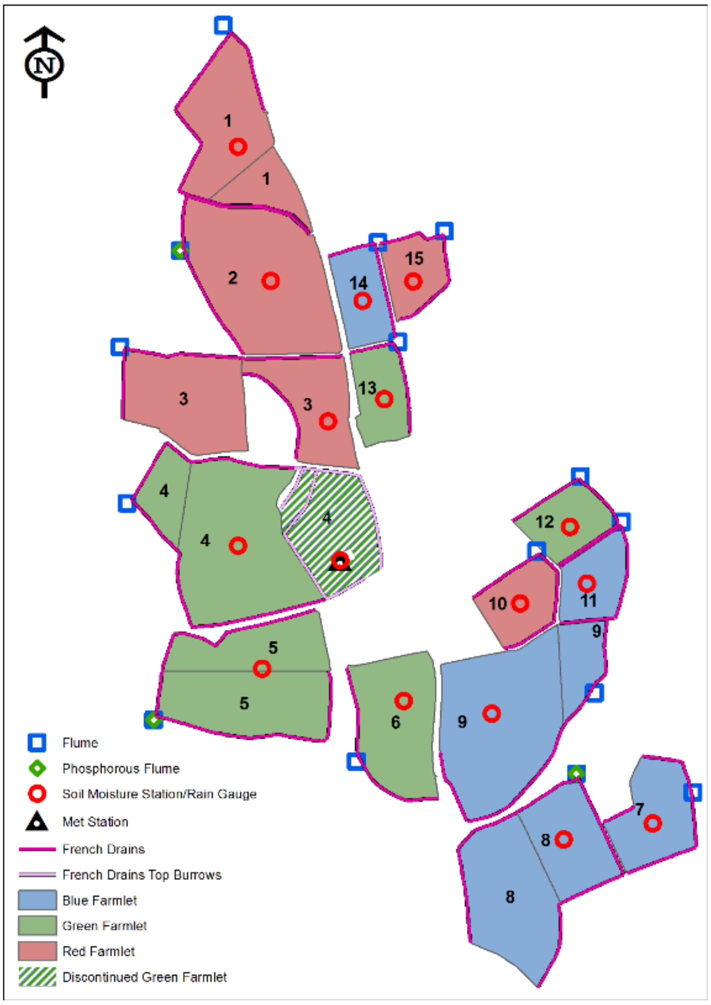

Figure S1. Map of the North Wyke Farm Platform in Devon, UK. Green = permanent pasture (PP); Blue = white clover/high sugar grass (WC); Red = high sugar grass monoculture (HS).

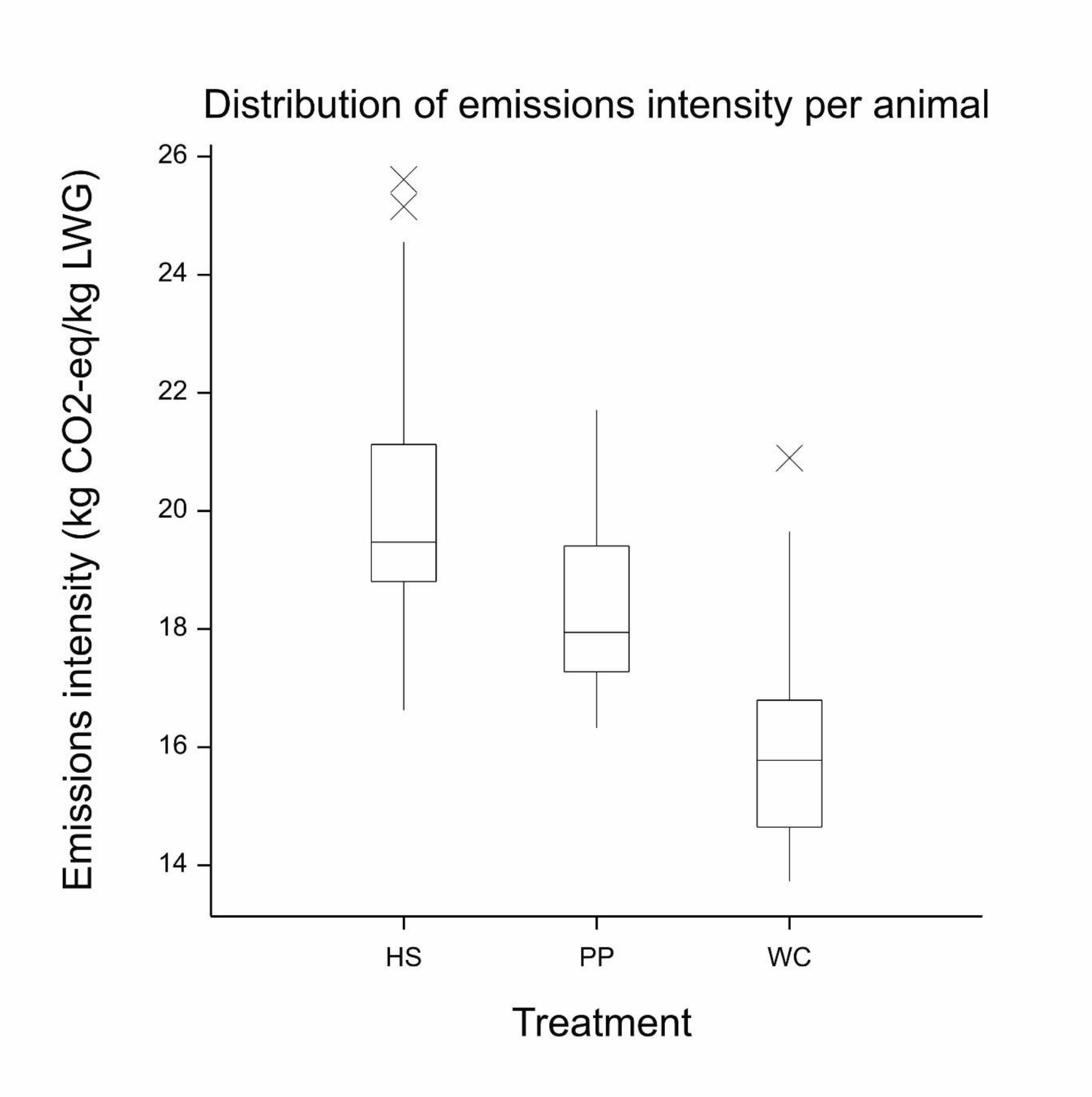

Figure S2. Distribution of emissions intensity per animal under each system. Outliers located further than 1.5 times the interquartile range beyond the quartiles are each denoted with a cross (×).

Table S1. Farm activities carried out under each system.

Activity	PP	WC	HS
Ploughing	✗	✓	✓
Rolling	✓	✓	✓
Harrowing	✗	✓	✓
Seeding	✗	✓	✓
Fertiliser spreading	✓	✓	✓
Herbicide spraying	✗	✓	✓
FYM spreading (solid)	✓	✓	✓
Liming	✗	✓	✓
Mowing	✓	✓	✓
Silage making	✓	✓	✓

Table S2. Distributions of uncertainty parameters assumed in Monte Carlo simulations.

Emission source	Uncertainty	Distribution	Reference
Animal/housing			IPCC (2006)
Methane (EF and MM)	± 20 %	Triangular	
Nitrous oxide (direct MM)	$SD^2 = 2$	Lognormal	
Nitrous oxide (indirect MM leaching)	-1500%/333%	Triangular	
Nitrous oxide (indirect MM volatilisation)	$SD^2 = 5$	Lognormal	
Pasture			IPCC (2006)
Nitrous oxide (direct)	$SD^2 = 3$	Lognormal	
Nitrous oxide (indirect leaching)	-1500%/333%	Triangular	
Nitrous oxide (indirect volatilisation)	$SD^2 = 5$	Lognormal	
Carbon dioxide (lime)	-50%/0%	Triangular	

Table S3. Comparison of emissions intensity (kg CO_2-eq/kg LWG) derived under two methods

System	Representative animal approach				Individual animal approach			
	Mean	LL[1]	UL[1]	Range	Mean	Min	Max	Range
PP	17.8	15.0	21.5	6.5	18.4	16.3	21.7	5.4
WC	14.4	12.7	16.2	3.5	16.0	13.7	20.9	7.2
HS	19.0	16.3	22.5	6.2	20.2	16.6	25.6	9.0

[1] Lower and upper limit values of the 95% confidence interval estimated by Monte Carlo simulations.
